# Importance of Heparan Sulfate Proteoglycans in Pancreatic Islets and β-Cells

**DOI:** 10.3390/ijms232012082

**Published:** 2022-10-11

**Authors:** Iwao Takahashi

**Affiliations:** Division of Molecular and Cellular Pharmacology, Department of Pathophysiology and Pharmacology, School of Pharmacy, Iwate Medical University, 1-1-1 Idaidori, Yahaba-cho, Shiwa-gun, Morioka 028-3694, Iwate, Japan; itakahas@iwate-med.ac.jp; Tel.: +81-19-651-5111

**Keywords:** diabetes melitus, pancreatic islets and β-cells, insulin secretion, heparan sulfate proteoglycans, core proteins, sulfotransferases, heparanase, signaling pathways

## Abstract

β-cells in the islets of Langerhans of the pancreas secrete insulin in response to the glucose concentration in the blood. When these pancreatic β-cells are damaged, diabetes develops through glucose intolerance caused by insufficient insulin secretion. High molecular weight polysaccharides, such as heparin and heparan sulfate (HS) proteoglycans, and HS-degrading enzymes, such as heparinase, participate in the protection, maintenance, and enhancement of the functions of pancreatic islets and β-cells, and the demand for studies on glycobiology within the field of diabetes research has increased. This review introduces the roles of complex glycoconjugates containing high molecular weight polysaccharides and their degrading enzymes in pancreatic islets and β-cells, including those obtained in studies conducted by us earlier. In addition, from the perspective of glycobiology, this study proposes the possibility of application to diabetes medicine.

## 1. Introduction

Diabetes mellitus (DM) is caused by a patient’s genetic background and environmental factors and develops when insulin secretion impairment and insulin resistance are intertwined [1,2,3,4,5]. The dysfunction of β-cells, which occupy most Langerhans islets in the pancreas, is one of the causes of insulin secretion failure, and research related to the analysis of insulin secretory function of pancreatic β-cells has contributed to the development of diabetes medicine [6]. However, it is still difficult to establish a glucose-stimulated insulin secretion (GSIS)-responsive cell line through inducing differentiation from embryonic stem/induced pluripotent stem cells. Further, multiple issues have yet to be addressed regarding the maintenance and improvement of GSIS function in isolated islets derived from organ donors. Several recent attempts to analyze the function of pancreatic β-cells from the viewpoint of glycobiology have been reported, and the connection between diverse roles of high-molecular-weight polysaccharides and the maintenance of islet homeostasis and insulin secretion has been analyzed [7,8,9,10,11,12,13,14,15,16,17]; this has increased the demand for glycobiology studies in the field of diabetes research.

High molecular weight polysaccharides have long been studied, and they have been analyzed in the eukaryotic development process [18] and in host infections by pathogenic microorganisms, such as *Chlamydia* spp. [19,20]. The role of polysaccharides has also been noted in adult mice and humans, and related research is gradually being conducted for medical applications. However, it is difficult to derive evidence-based medicine from these polysaccharides owing to their complex and diverse functions [21]. This review summarizes the studies linking the pancreatic islets of Langerhans and β-cells with high molecular weight polysaccharides and discusses their potential for usage in diabetes medicine.

## 2. Glycosaminoglycans with Heparin/Heparan Sulfate in Pancreatic Islets and β-Cells

Heparin and heparan sulfate (HS) are functional polysaccharides, and other linear sugar chains, such as chondroitin/dermatan sulfate (CS/DS), keratan sulfate (KS), and hyaluronan, are classified as glycosaminoglycans (GAGs) [22]. GAGs contain tens to hundreds of repeating units consisting of linear combinations of disaccharides composed of uronic acids (glucuronic acid, GlcA, and iduronic acid, IdoA) and amino sugars (glucosamine, GlcN; *N*-acetylglucosamine, GlcNAc; and *N*-acetylgalactosamine), and are classified according to their glycosidic linkage pattern and level of sulfation modification (Table 1) [23]. GAGs are biosynthesized in the Golgi apparatus via linker oligosaccharides attached to a core protein, except for hyaluronan [24,25]. Synthesized proteoglycans, which are complexes of core proteins and linear sugar chains, are found on cell surfaces and extracellular matrices and exert various physiological functions by regulating diverse signaling pathways, except for heparin [26]. Heparin has a higher IdoA content than GlcA and is stored in mast cell granules either bound to a core protein called serglycin or as free polysaccharide chains [27].

Heparin is a GAG that is more highly sulfated than HS and is widely used in the medical field for its anticoagulant effects. This substance has been utilized in diabetic medicine for many years to inhibit the complement cascade in an instant blood-mediated inflammatory reaction, which occurs mainly during islet transplantation [45,46]. Recently, to reduce the adverse effects of heparin administration throughout the body, a method to enhance islet viability by coating the islet surface with heparin was investigated [47,48,49]. The effects of heparin administration during islet transplantation include immunosuppression and the promotion of angiogenesis to transplanted islets through the fibroblast growth factor (FGF) signaling pathway. These actions improve the efficiency of islet engraftment following transplantation and maintain the function of β-cells.

Although the effective use of exogenous heparin in islet transplantation medicine is described above, the existence of acidic mucopolysaccharides, GAGs, in pancreatic β-cells has long been established endogenously [50]. Several studies have indirectly reported on the biological role of HS in pancreatic islets and β-cells. PI-88, which inhibits heparanase, an HS-degrading enzyme, may influence angiogenesis in spontaneous islet tumors in RIP-Tag2 mice [51], and HS proteoglycan (HSPG) in the basement membrane components of the islets may function as a physical barrier to protect pancreatic β-cells from attack by the inflammatory response in nonobese diabetic mice, which are a model of type 1 diabetes mellitus (T1DM) [52]. A genome-wide association study identified a haplotype block with a linkage disequilibrium that includes the *exostosin glycosyltransferase (EXT) 2* gene in type 2 diabetes mellitus (T2DM) patients [53]. *EXT2* encodes HS polymerase, mutations of which cause hereditary multiple osteochondroma [54,55]. The involvement of HS in pancreatic β-cells has been indirectly demonstrated using HS-degrading enzyme inhibitors, the expressions of HS core proteins, and bioinformatics, but at that time, there had been no research into the function or even the existence of HS in pancreatic β-cells.

We have demonstrated that HS is present in adult mouse pancreatic β-cells [7,8] using a 3G10 antibody, which recognizes an unsaturated disaccharide structure produced by the degradation of HS by a bacterial eliminase, i.e., heparitinase [56]. Furthermore, a specific deletion in mouse pancreatic β-cells of EXTL3, which influences HS synthesis, resulted in morphological abnormalities of the islets of Langerhans, decreased proliferative capacity of pancreatic β-cells, and impaired insulin secretion [7,8]. Concurrently, in mouse islets, the disaccharide content of CS and DS, the other GAGs of HS, were below the detection limits of the high-performance liquid chromatography (HPLC) [8,21] and immunostaining methods [14,30]. Although there are no reports of the detection of KS chains in pancreatic islets, the presence of lumican, a KS core protein, has been reported in human pancreatic α-cells and human pancreatic ductal adenocarcinoma cells [31,32,33,34,35,36]. Hyaluronan accumulates in islets as T1DM progresses and presents indirect β-cell destruction by inducing inflammation [37,38,39,40]. Moreover, in vitro studies have shown that hyaluronan directly impairs the insulin-secreting function of β-cells [41]. The effects of GAGs on pancreatic islets and β-cells are summarized in Table 1 [23]. Because CS, DS, and KS chains are undetectable in pancreatic β-cells and the presence of hyaluronan impairs pancreatic islets and β-cell function, heparin/HS is the only GAG potentially available to enhance the insulin-secreting function of pancreatic β-cells. Therefore, we focused our research on the role of HS in mouse pancreatic β-cells.

Since the author’s reports in 2009 [7,8], several research groups have analyzed the role of HS in pancreatic β-cells. In the pancreatic β-cells of spontaneous T1DM model mice, HS protects against the promotion of the autoimmune response to β-cell-destructive insulitis [12]. Furthermore, OGT2115, an inhibitor of heparanase, avoided a decrease in pancreatic β-cell HS and impaired insulin secretion in a mouse model of T1DM induced by streptozotocin (STZ), which causes pancreatic β-cell damage [17]. Moreover, insulin secretion is also reportedly impaired in hereditary multiple exostoses patients with mutations in the *EXT1* or *EXT2* genes [13], and a reduction of pancreatic β-cell HS in T1DM patients decreased the protection against hydrogen peroxide-induced β-cell death [15]. The expressions of the type XVIII collagen, syndecan-1 (SDC1), and CD44, which represent HSPGs, HS, and heparanase, respectively, decreased in islets from both young T2DM-prone db/db mice and Akita Ins2^WT/C96Y^ mice, in association with elevated endoplasmic reticulum stress markers [16]. In addition, in MIN6 cells and mouse and human β-cells, an HS mimetic also reduced hydrogen peroxide-induced cell death [12,15,16]. Therefore, a decrease in HS and HSPGs in pancreatic β-cells in DM leads to β-cell dysfunction, suggesting that inhibiting heparanase activity may protect pancreatic β-cell HS and thus inhibit DM progression.

## 3. HSPG Core Proteins in Pancreatic Islets and β-Cells

There are over a dozen core proteins of HS [26,57,58,59], and there have been several reports of the roles of core proteins in the islet basement membrane and pancreatic β-cells. Researchers suggested that type IV collagen and perlecan, which are among the components of the islet basement membrane in mice, influence the maintenance of lymphocytic infiltration (insulitis) and the major defense mechanisms of autoimmune diabetes in a mouse model of spontaneous T1DM [52]. These islet basement membrane components reportedly influenced the effector mechanism required for the graft rejection and rejection suppression needed for viability in mouse islet transplantation [60]. The deletion of perlecan in mice also reduces aggregations of islet amyloids, which are associated with losses of and dysfunction in β-cells, which is characteristic of T2DM [28]. In addition to the abovementioned type IV collagen and perlecan, the syndecan-4 (SDC4) expression was confirmed in intraislets, thereby suggesting that it influences pancreatic β-cell functions [30]. Collagen type XVIII, SDC1, and CD44 are also expressed in mouse pancreatic β-cells, and they may contribute to the antioxidant effects and increase in viability in transplanted pancreatic islets [29]. The expressions of the type XVIII collagen and SDC1 in T1DM patients, and a CD44 addition to the foregoing HSPGs in T2DM model mice, were reduced in pancreatic islets along with HS [15,16]. Recent findings using nano-liquid chromatography-tandem mass spectrometry method has reported that several proteins, including prohormones, not previously recognized as HSPGs, can become HSPGs in pancreatic β-cells [61]. However, there have only been a few studies on loss/gain in the functions of HSPG core proteins have provided the functional analyses of core proteins in pancreatic β-cells.

We subcloned MIN6 cells [62,63], which are derived from mouse pancreatic β-cells and have heterogeneous properties [9,10,64,65,66]; these subclones showed that the insulin secretory capacity correlates with the production of HS and the expression of the core protein SDC4 [10]. GSIS was impaired when *Sdc4* was knocked down (KD), and conversely, the SDC4 overexpression enhanced GSIS responsiveness with a significant increase in HS [10]. Thus, SDC4 represented an HSPG involved in GSIS functions in a study using cultured cells derived from mouse pancreatic β-cells.

SDC4 knockout (KO) mice have shown phenotypes, such as delayed skin wound healing, delayed angiogenesis in granulation tissue wounds [67], fetal vascular dysfunction in the placenta [68], and increased susceptibility to κ-carrageenan-induced renal injury [69]. Recently, we demonstrated abnormal glucose intolerance owing to impaired insulin secretion in a glucose tolerance test with 8-week-old male SDC4-KO mice of the C57BL/6J (B6) strain [70]. In contrast, the amount of HS in SDC4-KO islets was increased compared to that in wild-type islets [21,70]. Interestingly, these results suggest the possibility that HS bound to core proteins other than SDC4 may be unable to compensate for the insulin secretory function of HS with SDC4 as the core protein. In addition, no glucose intolerance was observed in 8-week-old male SDC4-KO mice of the Institute of Cancer Research (ICR) strain, which differs from the B6 strain [70]. In a mouse model of slowly progressive diabetes that does not show hyperglycemia until four weeks following low doses STZ administration [71], SDC4-KO mice showed hyperglycemia from four days after STZ administration and reduced casual insulin secretion and pancreatic β-cell mass [70]. Thus, STZ-induced damage to pancreatic β-cells appears to be more severe in SDC4-KO mice than in wild-type ICR mice. Hyperglycemia following pancreatic β-cell injury through STZ treatment suppresses the expression of the transcription factor PPARγ, resulting in the cancellation of the inhibition of heparanase expression by PPARγ and the promotion of HS degradation on pancreatic islets by heparanase [17]. STZ-treated SDC4-KO mice also showed an increased gene expression of heparanase compared to the control group, suggesting the existence of a molecular mechanism similar to that described above. However, because there are several reports of the positive [72,73,74,75] and negative [76,77,78,79,80] effects of PPARγ on islets and β-cell function, further analyses will be necessary to explain the increased susceptibility to STZ in SDC4-KO mice. These analyses of SDC4-KO mice revealed that SDC4 influences the insulin secretory function and the survival of pancreatic β-cells in cultured cells as well as in vivo in mice, although there are phenotypic differences in different mouse strains.

## 4. Sulfotransferases, Heparanases, Sulfatases, and Signaling Pathways in Pancreatic Islets and β-Cells

HS comprises a structure of repeating disaccharide units of uronic acid and GlcNAc, covalently linked to specific serine residues of the core protein via a tetrasaccharide (GlcA-galactose-galactose-xylose) linkage region. The repeating disaccharide region of HS is biosynthesized by the EXT family proteins at the Golgi apparatus; subsequently, the GlcNAc residues are deacetylated, and the resulting amino group is sulfated. Next, the GlcA in the flanking of the sulfated GlcN residue is epimerized to IdoA, and the hydroxy group at position 2 is sulfated. In addition, the hydroxy groups at positions 6 and 3 of GlcN can be sulfated [81,82,83]. HS chains of HSPGs transported from the Golgi apparatus to the cell surface can be fragmented and desulfated through the actions of heparanases and sulfatases [84,85].

HS on the cell surface and in the extracellular matrix interacts with different bioactive substances depending on their sequence pattern of isomerization and sulfation, which produces diversity in the functions that it exhibits [82,83,86,87,88,89,90,91,92]. FGF [93], transforming growth factor-β (TGF-β) [94,95], Wnt [96], and delta-like ligand (DLL)/Notch [97,98] influence signal transduction in pancreatic β-cells. These molecules bind to HS on the cell surface and are committed to regulating different signal transduction pathways [26], suggesting that HS influences the signaling of pancreatic β-cells. However, there is a paucity of reports suggesting a link between the sulfate groups of HS on pancreatic β-cells, signal transduction, and pancreatic β-cell function.

The detection of modified sulfate groups on HS chains in pancreatic islets has been analyzed through immunostaining and flow cytometry using various anti-HS antibodies. The 10E4 antibody recognizes *N*-acetylation/sulfation, RB4Ea12, and AO4B08 antibodies recognize *N*, 2-*O*-, 6-*O*-sulfation, and C5-epimerization, and the HepSS1 antibody recognizes continuous *N*-sulfation reacting in the β-cells of mice and human pancreases and the basement membrane of rats [12,14,15]. In contrast, EV3C3 and HS4E4 antibodies caused *N*-acetylation/sulfation, 2-*O*-sulfation, and C5-epimerization to react in α-cells of rat and human pancreas [14]. These analyses indicate the presence of highly sulfated HS in β-cells and low-sulfated HS in α-cells (Table 2). Interestingly, the 6-*O*-sulfation modification of HS and FGF receptors, which are abundant in β-cells, is absent in α-cells, and FGF1 and FGF2 are expressed at higher levels in α-cells than in β-cells [14]. Specifically, α-cells may act as paracrine FGF ligand suppliers in the FGF signaling pathway, which influences β-cell mass and the expression of *glucose transporter 2*(*Glut2*) and *prohormone converters 1/3* and *2*, which are characteristic of T2DM [14,93].

In an alternative approach to the methods described above, we used MIN6 cells, an inhibitor, sodium chlorate [99,100] for sulfation, and an interfering RNA against sulfate transferase to search for sulfate modifications to HS chains influencing the insulin secretory function. The results showed a compensatory increase in the gene expression of HS 3-*O*-sulfotransferase-1 (Hs3st1), which transfers a sulfate group to the hydroxy group at position 3 of GlcNAc in HS in sodium chlorate-treated MIN6 cells and impaired insulin secretion in Hs3st1 KD cells; this indicated that 3-*O*-sulfation influenced insulin secretory function in pancreatic β-cells [9]. The 3-*O*-sulfation performed by Hs3st1 has been reportedly been associated with blood coagulation by binding to antithrombin in vivo [101,102,103]. However, Hs3st2 is reportedly expressed only during daylight in the rat pineal gland, which controls the circadian rhythm [104]. HS modification by Hs3sts influences the proliferation of various cancer cells [105]; in *Caenorhabditis elegans,* Hs3sts are associated with neurite branching [106], and Hs3st3s influences the morphogenesis of fetal mouse salivary glands [107]. Infrequently, the phenotypes may not match in the 3-*O*-modification of HS. In *Drosophila*, a loss of Hs3st-B by KD will reportedly affect the stability or intracellular transport of Notch proteins [108]. However, Guo et al. reported that fruit flies with double KD in Hs3st-A and Hs3st-B showed no effect on Notch signaling [109]. Even though the 3-*O*-sulfate modification of HS is rare, there are as many as two to seven isoforms of the transferase in *Drosophila*, humans, and mice [110]; moreover, a loss of function of some Hsst isoforms is highly likely to be compensated by other Hssts [111,112,113]. However, regarding the involvement of Notch signaling in pancreatic β-cells, a recent study demonstrated that a blockade of DLL/Notch signaling by antibodies against DLL4 protected islets and β-cell homeostasis and reversed diabetes in nonobese diabetic mice (T1DM spontaneous model) and STZ-treated mice and promoted differentiation and proliferation from pancreatic progenitor cells to insulin-producing cells in wild-type mice [97]. Furthermore, DLL/Notch signaling is reportedly essential for maintaining pancreatic β-cell function homeostasis, including insulin secretion using DLL1 and DLL4 deficiencies and a DLL1 overexpression [98]. We have also reported that KD of the *Hs3st1* gene [9] causes an expression of the *Dll4* gene in cell culture systems [114], although the possibility that this is caused by an off-target effect cannot be excluded. Further analyses utilizing loss-of-function/gain-of-function evaluations through gene knockout are needed. 

Although the analyses of the 3-*O*-sulfation modification structures of HS are more difficult than those of other sulfated modifications, an HS4C3 antibody that recognizes antithrombin (AT)-binding 3-*O*-sulfate groups on HS [115] and disaccharide and tetrasaccharide composition analyses, including non-AT-binding 3-*O*-sulfated units using reverse-phase ion-pair HPLC with a post-column fluorescent labeling system [116], have been developed. Future multidimensional analyses of various sulfate groups, including 3-*O*-sulfation of HS [117], may reveal additional signaling pathways linking HS sulfate groups to pancreatic β-cell functions.

Heparanase was identified by cDNA cloning as the only mammalian endo-β-D-glucuronidase that degrades HS [118,119,120,121,122]. The discovery of heparanase is well-established, and since 1975, when its activity was confirmed in a rat liver lysosomal fraction [123], HS-degrading activity has been observed in fibroblasts, mast cells, and platelets, and there is a correlation between the metastatic potential of malignant tumor cells and their HS-degrading activity in the basement membrane [124]. Heparanase also assists in regulating HSPG turnover in normal cells, degrading HSPGs that are barriers to cell migration in basement membranes and extracellular matrix, and promoting leukocyte migration from blood to inflammatory sites [125,126,127,128,129,130,131]. 

**Figure 1:**
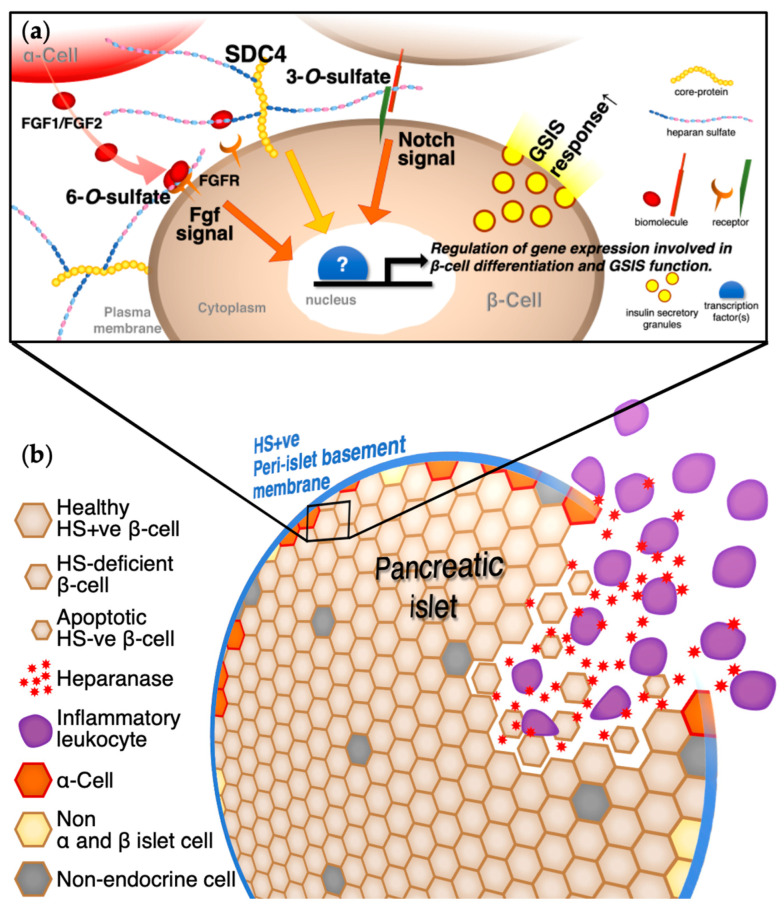
Schematic diagram of the actions of heparan sulfate proteoglycans (HSPGs) and related molecules on pancreatic islets and β-cells. (**a**) The actions of HSPGs in pancreatic β-cells should be considered on the heparan sulfate (HS) side bound to the core protein as well as via the intra- and extracellular domains of the transmembrane syndecan-4 (SDC4) core protein. These molecular mechanisms downstream of HSPGs appear to regulate gene expression involved in pancreatic β-cell differentiation and glucose-stimulated insulin secretion (GSIS) function and contribute to the maintenance and enhancement of GSIS-responsive function. (**b**) Multistep impairment by heparanase from inflammatory leukocytes against the HS of pancreatic islets and β-cells as one of several factors in the development of diabetes. Prolonged hyperglycemia also adds to the damage caused by heparanase from pancreatic β-cells, causing diabetes to increase in severity.

Diabetes progression and inflammation are closely related [132,133,134,135], and the involvement of heparanase in islet and β-cell damage during diabetes progression in human diabetics and model mice has been investigated in detail by Simeonovic et al. [12,15,16,52]. The process heparanase influencing the progression of T1DM is as follows: first, HS in the peri-islet basement membrane degrades by heparanase produced by migrated inflammatory leukocytes from the vasculature to the pancreatic islets; damage to the islet basement membrane barrier then causes inflammatory leukocytes to invade the intraislets. Thus, heparanase is locally produced and degrades intra-β-cell HS, resulting in a decrease in overall islet β-cell HS and an increase in β-cell death and leading to a deficiency in insulin production capacity and the development of T1DM [136,137] (Figure 1). In STZ-treated mice, the heparanase expression was also elicited by a decreased expression of PPARγ, which suppressed heparanase transcription, when pancreatic β-cells were exposed to a hyperglycemic environment [17]. Therefore, as hyperglycemia is prolonged, heparanase is likely to cause increasingly severe pancreatic β-cell injuries. Thus, one of the strategies to treat diabetes is to inhibit the multistep impairment of heparanase to the HS of pancreatic islets and β-cells, which is one of the factors in the development of diabetes.

The sulfatase group responsible for the desulfation of HS includes extracellular sulfatases, except for the lysosome-localized type, which regulates HS signaling functions [138,139,140]. These sulfatases release the 6-*O* sulfate group from IdoA2S-GlcNS6S or GlcA/IdoA-GlcNS6S units [140,141,142,143]. Extracellular sulfatases have reportedly regulated signaling through regulating the binding of HS/heparin-binding factors, such as the glial-cell-line-derived neurotrophic factor [85], bone morphogenic proteins [143], Wnt [138,142,144], FGF [145], and TGF-β [146,147] to the HS sugar chains. Although there are few reports on the involvement of extracellular sulfatases in islets and β-cells, FGF [93], TGF-β [94,95], and Wnt [96] have reportedly influenced pancreatic β-cell functions, and new findings linking extracellular sulfatases and pancreatic β-cell function may be obtained in the future.

## 5. Conclusions

The roles of HS, core proteins of HSPGs, sulfation modifications related to signaling pathways, and heparanase in pancreatic islets and the β-cells introduced in this review are summarized in Figure 1. Considering the actions of HSPGs in pancreatic β-cells, it is necessary to consider both the action of the extracellular domain to which HS sugar chains bind as well as the action via the intracellular domain of the core protein in the case of SDC4 and others [148,149,150,151]. The roles of HSPGs other than SDC4 have also been analyzed extensively, and the biological positions of HSPGs have been elucidated in pancreatic islets and β-cells [15,28,29,30,52,60]. 

Based on the roles of HSPGs in pancreatic islets and β-cells, the medical treatment of diabetes could be enhanced using HSPGs as target molecules. PI-88, OGT2115, and heparinoids (functional HS) are candidates for diabetes drugs targeting HS because they have HS-like structures and maintain the functions of pancreatic islets and β-cells by compensating for an impaired HS function or by inhibiting heparanase [12,17,152,153,154,155]. In addition, GAGs with sulfate groups and branching modified at new sites that have not been reported in vertebrates were discovered in marine invertebrates [42,43,44,156]. This diversity of the modifications of marine GAGs may render them as helpful medical HS/heparin analogs in the future. However, as the heparin treatment promotes the fibrosis of amyloid-forming proteins during islet transplantation, HS/heparin analogs also risk promoting islet amyloid aggregation [157,158].

Controlling insulin secretory function by regulating the *Sdc4* gene expression may also be a target for diabetes therapy [10,159]. We have identified a *cis*-element region on the promoter of the *Sdc4* gene and have found compounds that upregulate *Sdc4* gene expression in a region-dependent manner [159]. Because cells treated with this compound trichostatin-A showed enhanced GSIS responsiveness [159], it is currently under investigation as a candidate molecule for developing diabetes drugs.

In addition, in FGF signaling, which also influences pancreatic β-cell functions, PG-FGF-1 (HS-modified FGF1), a chimeric molecule of FGF1 and SDC4 [160], and FGF-C, a chimeric molecule of FGF1 and FGF2 [161], are reportedly more effective than FGFs (natural ligands for FGF receptors) in wound-healing and radioprotection [162,163,164]. This direction of application of molecules that regulate signal transduction to diabetes care may also be useful.

Research on glycobiology in pancreatic islets and β-cells has continuously developed, but there are still a few topics of considerable interest that have yet to be explained. These include determining why pancreatic β-cells synthesize more HS than other GAGs [8,14,21,30], determining why other HSPGs are unable to compensate for the function of SDC4 [10,70], and within GSIS functioning, determining which bioactive substances interact with which sulfate groups on HS and thereby designating which signal is regulated by HS. Additional research in this field will hopefully answer these and any further questions that may arise in the future.

## Figures and Tables

**Table 1:** Features affected by GAGs in pancreatic islets and β-cells.

Disaccharide Structures	Link between GAGs and Pancreatic Islets and β-Cells
Heparin/Heparan sulfate (HS) 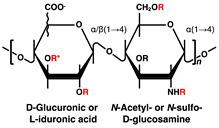	Impaired postnatal islet growth, β-cell differentiation, and insulin secretion in HS biosynthesis enzyme knock out mice [7,8,9,10,11]. Islet-protective effects of heparanase inhibitors or HS in diabetic mouse models (avoidance of β-cell death and HS loss through the inhibition of heparanase activity) [12,17]. Contribution to β-cell mass and insulin secretion capacity in hereditary multiple exostosis subjects [13]. Different sulfate modification pattern features HS in α-cells and HS in β-cells of rat and human pancreatic islets [14]. Loss of HS and the heparanase expression through islet-infiltrating leukocytes in human type 1 diabetes mellitus (T1DM) islets [15]. Reduction of islet amyloid deposition by perlecan depletion in human islet amyloid polypeptide transgenic mice [28]. Contribution of HS to antioxidant activity and viability increased in transplanted islets [29].
Chondroitin/dermatan sulfate (CS/DS) 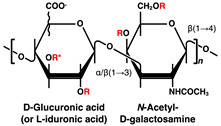	CS/DS is below the detection limit by high-performance liquid chromatography and immunostaining methods in mouse and rat islets [8,14,21,30].
Keratan sulfate (KS) 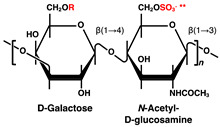	No reports of detection of KS chains in pancreatic islets to date. Lumican, a KS core-protein, is expressed in human pancreaticα- and human pancreatic ductal adenocarcinoma cells [31,32,33,34,35,36].
Hyaluronan (HA) 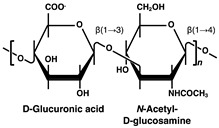	HA accumulation in pancreatic islets with T1DM progressing and the following inflammation and β-cell destruction [37,38,39,40]. HA directly impaired in the insulin secretory function of β-cells in vitro [41].

R = –H or –SO_3_H. At the R* position, sulfation was discovered in bivalve HS [42], and α-L-fucose branching has been reported in sea cucumber [43] and king crab CS [44]. ** *N*-acetylglucosamine is normally 6-*O*-sulfated.

**Table 2:** GAG domain-specific antibodies, binding loci in pancreatic islets, and modifications for enhancing their binding.

Antibody	Binding Sites of Antibodies in Pancreatic Islets	HS Modifications Required for Antibody Binding
10E4	Mouse intra-islet-β-cells [12], human intra-β-cells [14,15], and rat islets basement membranes [14]	*N*-acetylation/sulfation
RB4EA12	Human and rat intra-β-cells [14]	*N*-sulfation, 2-*O*-, 6-*O*-, C5-epimerization
AO4B08	Human and rat intra-β-cells [14]	*N*-acetylation/sulfation, 6-*O*-sulfation
HepSS-1	Mouse islet-β-cell surface [12]	*N*-sulfation
EV3C3 *	Human and rat α-cells [14]	*N*-sulfation, 2-*O*-sulfation, C5-epimerization
HS4E4 **	Human and rat α-cells [14]	*N*-acetylation/sulfation, C5-epimerization
HS4C3	Nuclei of the cells [14]	*N*-sulfation, 2-*O*-, 6-*O*-, 3-*O*-sulfation

High 6-*O*-sulfation * and 2-*O*-, 6-*O*-sulfation ** may reduce the binding of the respective antibodies.

## Data Availability

Not applicable.
